# An Experimental Test Proposal to Study Human Behaviour in Fires Using Virtual Environments

**DOI:** 10.3390/s20123607

**Published:** 2020-06-26

**Authors:** Carlos de Lama, Cristina González-Gaya, Alberto Sánchez-Lite

**Affiliations:** 1 Department of Construction and Manufacturing Engineering, ETSII, National Distance Education University (UNED), C/Juan del Rosal 12, 28040 Madrid, Spain; cdelama2@alumno.uned.es (C.d.L.); cggaya@ind.uned.es (C.G.-G.); 2 Department of Materials Science and Metallurgical Engineering, Graphic Expression in Engineering, Cartographic Engineering, Geodesy and Photogrammetry, Mechanical Engineering and Manufacturing Processes Engineering, School of Industrial Engineering, University of Valladolid, Paseo del Cauce 59, 47011 Valladolid, Spain

**Keywords:** virtual environment, behavior, fire, evacuation

## Abstract

Human behavior in an emergency situation is the starting point for all evacuation planning projects. A better understanding of the decisions made by the occupants during an emergency can help to develop calculation tools that can create more efficient forms of visual and audio communication and implement better procedures for evacuating people. The difficulty in studying human behavior lies in the very nature of emergencies, as they are unpredictable, somewhat exceptional and not reproducible. Fire drills play a role in training emergency teams and building occupants, but they cannot be used to collect real data on people’s behavior unless the drill is so realistic that it could endanger the occupants’ safety. In the procedure described here, through the use of a Virtual Reality device that encompasses all critical phases, including user characterization data before the virtual experience, building design parameters and fire scenario, key variables of human behavior can be recorded in order to evaluate each user’s experience satisfactorily. This research shows that the average delay in starting an evacuation is greater than one minute, that anxiety levels and heart rates increase during a fire and that people do not pay attention to evacuation signals. Further analysis of the quantitative data may also provide the causes for decision-making. The use of devices that create realistic virtual environments is a solution for conducting “what if” tests to study and record the decisions taken by the users who undergo the experience in a way that is completely safe for them.

## 1. Introduction

The use of simulators and virtual environments for the visualization and study of the behavior of complex systems is applied in many scientific fields [1,2,3,4,5,6,7,8,9].

The use of devices that create realistic virtual environments allows the researcher to record behavioral data, in order to successfully evaluate each user’s experience. These realistic virtual environments could be used due to the impossibility of capturing and recording real-time behavior data in real fire situations, given the nature of these types of emergencies, which are unpredictable, not reproducible and infrequent.

The decisions made by people in emergencies have been studied for decades. Kobes et al. [10] reviewed the available information, up to the date of publication, on human behavior, in the case of fire, insofar as building safety is concerned, making a general assessment of the critical factors that determine occupants’ conduct depending on the environment, fire dynamics and human behavior.

The information available thus far has usually been obtained by two methods: analyzing the flow of occupants from a building where a fire drill is taking place, with movements being recorded by various techniques, such as capturing images or arranging observers, or post-fire interviews with the survivors of a real incident.

It is difficult to obtain reliable data with either of these methods. Fire drills do not offer the right conditions for creating a user environment in which most of the behavioral parameters, such as smoke, are relevant.

As far as interviews with fire survivors or emergency teams are concerned, the data are obtained through subsequent interviews and may therefore be influenced by memories or sensations.

In this regard, Haghani and Sarvi [11] analyze how occupants leave a building, placing different priorities on the information they receive, depending on whether or not there is a real emergency and, consequently, how their decisions change. This process of prioritizing information is influenced by the length of the escape route, the number of occupants, the visibility of the exits or the impact which the decisions of some occupants have on the rest of the group.

This topic was also studied by Cordeiro et al. [12] In their study, human behavior was analyzed by using questionnaires or personal interviews with survivors who have lived through different emergency situations.

As for obtaining behavioral data from the point of view of the rescue teams, when they took a survey of UK firefighters, Hulse and Galea [13] found that these professionals are not immune to experiencing states of anxiety about perceived risks, although they are apparently psychologically prepared to do the job they are trained to do.

With regard to simulations or controlled experiments where behavioral data can be obtained, there are studies such as the one conducted by Fridolf et al. [14], who used controlled experiments to analyze the decisions taken by the occupants in a very specific emergency situation, such as the evacuation of a tunnel.

Another interesting type of study that uses simulations is the analysis of behavior from a different point of view, such as the occupants’ perception of their environment. Castel et al. [15] analyzed the willingness of the occupants of a building to memorize where certain items, such as fire extinguishers, are located, in case they should need them if there is a fire.

Another example of a simulation used to obtain data is that of Fridolf et al. [16], whose study analyzed evacuation speed through smoke, with the values obtained varying according to the extinguishing coefficient. This shows that an element that is present in a fire and cannot be reproduced in evacuation drills has a negative impact on evacuation speed.

People’s physical activity can be analyzed by other methods. The use of wearable motion-sensing technology offers important advantages over conventional methods for obtaining measures of physical activity and/or physical functioning in individuals with chronic diseases, with reduced mobility or with disabilities. Allet et al. [17] identified the actual state of applying wearable systems for monitoring mobility-related activity in individuals with chronic disease conditions.

The use of Virtual Reality (VR) as a method to study behavior is still in its early stages. Virtual Reality is the use of computer technology to create an immersive three-dimensional environment.

The first research to use Virtual Reality to analyze human behavior was for therapeutic purposes in the case of phobias. In Reference [18], the authors’ goal was to examine the efficacy of Virtual Reality graded exposure in the treatment of acrophobia (fear of heights).

Virtual Reality and interactive video gaming have emerged as recent treatment approaches in stroke rehabilitation, with commercial gaming consoles, in particular, being rapidly adopted in clinical settings. Laver et al. [19] determined the efficacy of Virtual Reality compared with an alternative intervention.

Virtual Reality was also used with people with Parkinson’s disease [20]. The objective was to determine the effect of Virtual Reality training on walking and balance and to analyze the effects of VR on overall motor function, activities of daily living, behavior and decision-making.

Virtual environments give researchers the ability to bring in training scenarios that are not feasible or are potentially risky to recreate in the real world. Nickel et al. [21] analyzed whether the use of a Virtual Reality tutorial session before using a certain mining simulator could increase the consistency of the performance metrics of the participants.

Kinateder et al. [22] analyzed the strengths and weaknesses, as well as the opportunities and threats, of Virtual Reality as a research tool, knowing that this methodology can replicate different aspects of a fire, without risk to the user. They came to the conclusion that virtual environments provide a maximum of experimental control, are easy to replicate, have a relatively high validity and allow for the safe study of occupant behavior in scenarios that otherwise would be too dangerous.

Virtual Reality has also been a useful tool for treating certain behavioral disorders. Kritikos et al. [23] evaluated the feeling of presence during different hardware setups of Virtual Reality exposure therapy and, particularly, how the user’s interaction with those setups can affect their sense of presence during the virtual simulation.

Examples of research using the Virtual Reality methodology include the study by Ren et al. [24], who applied this technology to disaster evacuation simulation. In this case, the results showed that Virtual Reality was a valid method that can be used for evacuation simulation and building safety.

Another example is the study by Gamberini et al. [25] In this study, users encountered different emergency scenarios with varying degrees of intensity. The results showed that, once the emergency began, it caused significant changes in people’s movements. It can therefore be inferred that a virtual risk situation will produce adaptive responses and that Virtual Reality may be suitable for emergency simulations.

Taking another approach, it is possible to analyze the behavior of victims of past fires by recreating known fatal fires. In their study, Arias et al. [26] found that those who participated in the experience made decisions based on the fire scenario in which they were immersed, displaying a certain level of stress due to the simulated emergency, despite knowing that the threat was not real.

The use of wearable motion-sensing technology offers important advantages over conventional methods for obtaining measures of physical activity and/or physical functioning in individuals with chronic diseases.

Similar technologies have been used for the analysis of the Human Activity Recognition (HAR) that refers to an emerging area of interest for medical, military and security applications [27], or studies that classify and model various human activities in a supervised lab-based protocol and utilize the model to identify physical activity in a free-living setting [28].

In this research, one of the objectives was to use virtual environments to learn about people’s decision-making in the event of a fire. Virtual environments, which are already well-known, have been used for the field of building construction and reconstruction. The aim of this paper is to show a protocol to record behavioral parameters in case of fire.

Human behavior data can be obtained by using a variety of research methods and data collection techniques. It is important to understand how the data were obtained, since the choice of research method and data collection techniques can influence the validity and reliability of the results.

Data collection techniques commonly used in human behavior in fire research can be broadly categorized as surveys, observations and simulations [29].

Surveys: data collected through questionnaires or interviews designed to determine the characteristics, actions, opinions, etc., of a particular sample.

Observations: Movement/actions are directly observed in some form, without necessarily relying on verbal communication with those involved. Observation can be done by stationary video cameras, roving video cameras or human observers.

Simulations: Computer tools cannot be used to analyze a fire in real time. Table 1 shows a comparative overview of the different techniques.

This protocol is a predefined written procedural method for obtaining human behavioral data in case of fire.

The protocol proposed in this article establishes which resources and which procedures are most likely to result in behavioral data that can be easily analyzed in any scenario and any fire, considering all the parameters that can modify behavior during the time that the evacuation lasts.

The tests were carried out for the particular case of a fire, but this protocol can easily be adapted to other types of emergencies, by developing new virtual environments.

## 2. Materials and Methods

### 2.1. Virtual Environment

The materials and devices described in this section were used to capture and record user behavioral data:

An HTC Vive Virtual Reality device was used to generate a virtual environment and to capture the behavioral data. Table 2 shows the hardware specifications [30,31,32].

**Figure 1:**
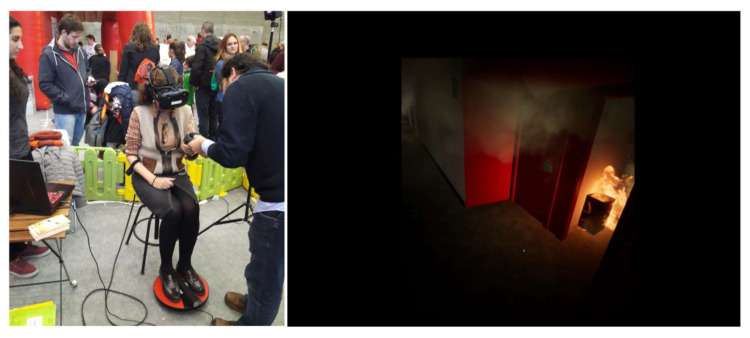
User experience and virtual environment.

**Figure 2:**
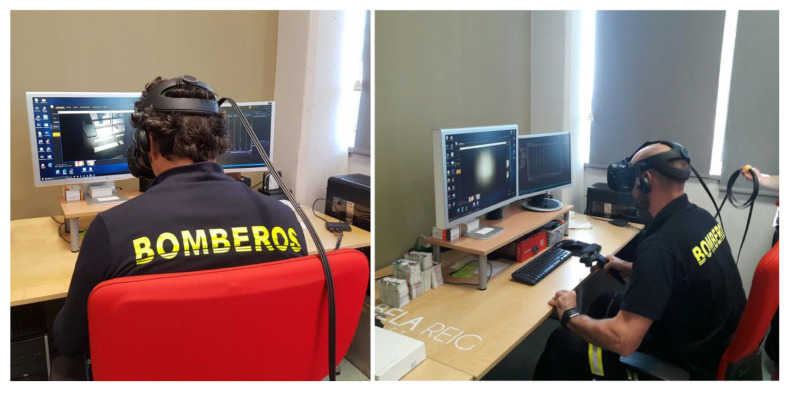
Firefighter tests.

Figure 1 shows a user experience and the virtual environment. Figure 2 shows the firefighter testers who certified the realism of the fire in the virtual environment.

### 2.2. Methodological Approach

The method described in this approach is valid as long as there is a Virtual Reality device and the appropriate software to generate the virtual environment and the emergency to be analyzed. The protocol described in this section defines the steps to be followed for the configuration of the measurement system, along with the user’s setup and instrumentation.

The following parameters were considered as the most representative to analyze the decision-making of a person during an emergency:

- Recognition time: interval between the time when the alarm sounds and the time when the user realizes there is an emergency.
- Response time: interval between the “recognition time” and the time when the user begins to move to a safe zone.
- Travel time: interval between the “response time” and the moment when the user arrives at a safe zone.
- Complete trajectory from the time the alarm sounds to the end, identifying the trajectories in each evacuation phase.
- Speeds at each point along the trajectory, including the places where the user crouches or squats due to the effects of smoke during the evacuation.
- Visualization of emergency signals, detecting which signals are seen by the user and at what point of the experience.
- Sudden head movements.
- Heart rate.

The data obtained cover the user’s behavior from the time the alarm sounds until he or she reaches a safe zone, and can be compared with the user’s behavior before the incident occurs.

**Figure 3:**
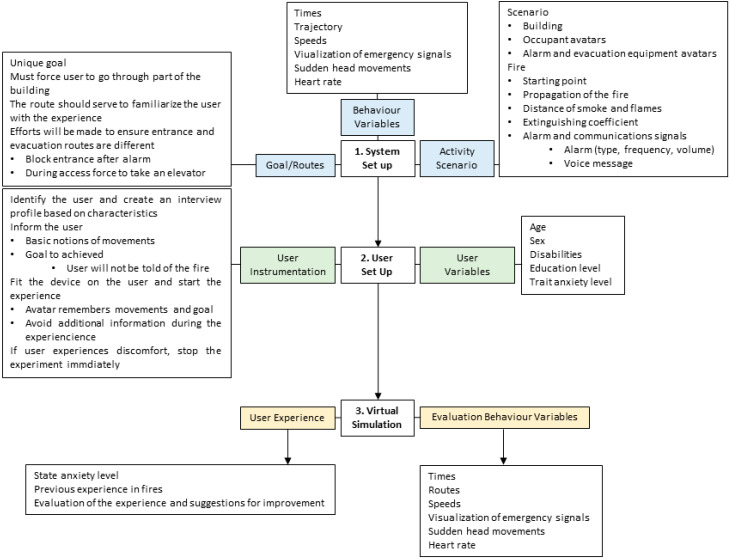
Outline for the implementation of the protocol.

The analysis of the data can help design buildings, fire protection systems and evacuation procedures, with the goal of minimizing harm to people in the event of a fire. Behavioral analysis is also suitable for training emergency response teams and for the specific analysis of audio–visual alarms and communications. Figure 3 shows the protocol for obtaining response parameters for the human behavior study in emergency situations, using virtual environments. It consists of seven steps:

Step 1: Establish a method to identify the user, including the characteristics of his/her profile that are considered relevant to the research by conducting an interview.

Some user profile characteristics are identified as an example. Characteristics may vary depending on the research objectives. The protocol proposes, among others, the following parameters: age, sex, anthropometry, disabilities (motor, sensory and cognitive), education level and trait anxiety level.

The level of trait anxiety is determined by administering a State-Trait Anxiety Inventory (STAI) questionnaire before the experiment, without telling the user what the purpose is.

Step 2: List the behavior variables to be recorded.

Behavioral data are captured and recorded in real time, through the Virtual Reality device. Characteristics may vary depending on the research objectives. Seven behavioral variables are identified in Table 3, as an example:

Step 3: Create the design and conditions of the scenario and the activity within the scenario.

This step includes designing the building, determining the number of virtual occupants (avatars) inside the building and the activities they perform during the experience (different types of behavior can be programmed), and determining the number of avatars that act as alarm and evacuation equipment (these avatars can perform communication and assistance functions and help with the evacuation process).

Step 4: Design the fire scenario.

In this step, the following parameters are defined: starting point of the fire, propagation of the fire according to interior design, building materials and passive protection (flames and smoke), minimum distance at which the smoke and flames affect the user and smoke stratification (this information can be provided by firefighters, the extinguishing coefficient for smoke, the opacity of the atmosphere is a consequence of the amount of solid particles in the air, which makes it difficult to see objects depending on how far away from the user they are), type of alarm and communication signals for users, type, frequency and volume of the fire alarm, alarm voice message (the alarm message must be set up to be effective, with a total duration of less than 30 s, divided into three phases: call to attention, reason for the call and information on what to do).

Step 5: Set a goal to be achieved by the user, as part of the experience.

The goal will be unique for all users, e.g., locate a package in an office of an administrative building, and must be such that it forces the user to walk through part of the building.

The route that the user must take to achieve the goal must be long enough to serve as a learning experience and to familiarize the user with moving around the virtual environment. The entrance route to the building should be different than the evacuation route. There are different options for meeting this requirement. The entrance can be blocked once the fire breaks out, in order to force a different evacuation route. If the scenario has more than one floor, force the user to take an elevator to achieve the goal. Once the fire starts, the elevator is blocked and cannot be used for evacuation.

Step 6: Determine user status and characteristics.

In this step, we conduct interviews after the experiment, to determine user status and characteristics. The protocol identifies state anxiety level (this can be determined by means of a STAI test after the experiment) and previous experience in fires.

The State-Trait Anxiety Inventory (STAI) is an instrument that quantifies adult anxiety (a children’s version is also available). This particular instrument is used to simplify the separation between state anxiety and trait anxiety and feelings of anxiety [33,34,35].

The questionnaire in this research was simplified and adapted for the users of Virtual Reality. The aim of the questionnaire is not to quantify the increase in anxiety, but to check if there is a difference between the trait anxiety and state anxiety of the users.

Step 7: Outline the procedure for the user to undergo the experience.

First, the user is identified, and his/her profile is created, as seen in Steps 1 and 2 of the protocol. Then, the user is provided with the necessary information to carry out the experience. This means basic notions of how to move around in a virtual environment. The user becomes familiar with these movements during the experience.

Finally, the detailed information on the goal to be achieved is loaded into the system (the user is not given any information about the event that will occur during the experience), and we provide the user with a Virtual Reality helmet (sensory, visual and auditory immersion) and any other available accessories, such as hand controls, foot platforms, or heart rate or blood pressure sensors.

The equipment was calibrated before the user experiences. The Virtual Reality device includes a driver for calibration. The research group calibrated the device and supervised 100% of the experiences.

When the experience begins, an avatar can remind the user of the basic notions of movement, as well as the goal, in such a way as to familiarize the user with the environment. Once the experience has begun, the user cannot be helped to locate the target, such as by voices or superimposed virtual arrows, and may only be guided by the building’s signals. If the user experiences any kind of dizziness or discomfort, stop the experiment immediately.

Figure 3 shows an outline for the implementation of the protocol, including the phases of the user’s experience.

## 3. Results and Discussion

The HTC Vive Series Virtual Reality system uses a Virtual Reality headset to generate realistic images and sounds that simulate the physical presence of a user in a virtual environment. Users can look at the artificial world, move around in it and interact with virtual features or elements. The office ambience sounds, the sound of the alarm and the realistic sound of the fire are essential within the virtual environment.

The protocol was evaluated with a single virtual environment in which 300 users of different profiles participated. It was supervised and validated by experts from the professional association of fire technicians, with a complete and usable model. These experts advised the research group during the development of the virtual environment. Users ranged in age from 18 to 74. In total, 42% of users were women, and 58% were men. Table 4 shows the users’ ages.

A description of a representative case is provided in Figure 1, to analyze the results of the use of this protocol. Table 5 and Table 6 resume the user profile data and the user behavioral data of representative cases.

A questionnaire with six questions was carried out, to analyze the level of users’ trait anxiety before the experience. The possible answers are 1 to 4, with 1 being the lowest and 4 the highest. The questions were as follows: Are you calm? Do you feel safe? Do you feel comfortable? Are you worried? Do I have confidence in myself? Is this the first time you have done this test?

The information provided to the user was as follows:

- Movements that are possible in the virtual environment (walking, running, turning, looking around or bending).
- Mission within the virtual environment: The user must locate a package in office 6-A, on the first floor of the building.

The user is free to move around within the virtual environment. The user can choose the path within the building and move his or her head in order to visualize what might interest him or her.

**Figure 4:**
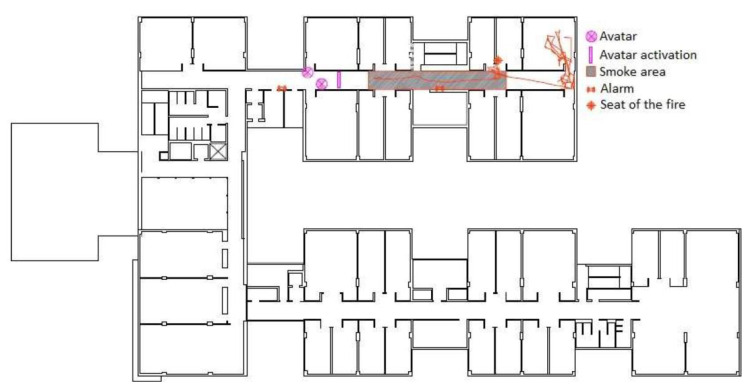
The red line shows the evacuation route.

The recording of behavioral parameters begins once the alarm sounds. The results were as follows:

- Recognition time: 0.1 s.
- Response time: 38 s.
- Travel time: 93 s.
- Visualization of evacuation signals: User does not see any evacuation signs.
- Takes the path indicated in the drawing.
- Average heart rate before the alarm signal: 78 beats per minute.
- Average heart rate 30 s after alarm sounds: 83 beats per minute, 7% increase.
- Average heart rate while user crosses the area filled with smoke: 104 beats per minute, 6% increase.
- Increase in the number of sudden head movements in the same section of the evacuation route before and after the alarm sounds, with the difference being from one movement before the alarm to 31 movements along the same stretch, once the alarm sounds.
- The path taken by the user is reflected in Figure 4.

Once the experience is over, a new questionnaire with eight questions was administered to the user. These questions were as follows: Are you calm? Do you feel safe? Do you feel comfortable? Are you worried? Do I have confidence in myself? To what extent do you think your level of anxiety increased during the experience? Have you ever experienced a real fire in a building? Do you think experiences like this can help people’s safety?

Given the complexity of human behavior, partial conclusions can be drawn about decision-making during the experience, and, in this way, an attempt can be made to establish a behavioral profile.

With this protocol, it is possible to establish the following:

- Differences between behavior before and after the alarm signal.
- Differences between the recognition and response time intervals and the evacuation time.
- Whether the user experiences behavioral changes, such as rapid eye movement and changes in heart rate.
- Whether the user sees evacuation signs.
- Depending on the route taken during the experiment, parameters can be set to assess the user’s perception of danger.

With the recorded data, it is possible to analyze different combinations in order to obtain different conclusions, depending on the researcher’s objective.

Because data logging is so highly automated, there are no critical steps that can hinder the success of the user experience.

The specifications of the device mean that the experiment is ideally performed on healthy individuals, independently. At the beginning of the experiment, the sensors must be checked to make sure they are all working properly.

A small number of users were unable to finish the experience due to motion sickness or disorders caused by a lack of coordination between movement that is perceived visually and the direction of movement of the vestibular system, where sensory information related to the control of balance and ocular movement is processed. The symptoms they experienced include dizziness and, in very rare cases, nausea that disappears within a few minutes once the experiment is stopped.

The system records the user’s behavior, without the user being aware that data are being generated.

In all, 100% of the users who completed the experience acknowledged, due to the immersion experience, that the decision-making in a real fire would have been very similar or identical to the decision-making in the simulation.

The realism of the virtual environment and experience was validated by experts from the fire department, as well as user feedback after the experience.

The objective of this protocol is to obtain the behavioral data in a single scenario, but this protocol is easily adapted to other scenarios.

All users, including firefighters, who completed the experience rated this system as a very useful tool for fire training.

The increase in the user’s heart rate and the number of head movements, once the virtual fire starts, demonstrates that behavior varies before and after the alarm.

The possibility of having other sensations such as the smell of smoke or heat was analyzed. One of the experience requirements is that the user does not know that there will be a fire. Smoke or heat can be perceived clues to the next user, so it was decided to incorporate this type of stimulus in further developments of the experience.

An improvement proposal is the use of augmented reality to particularize cases in specific buildings. Capturing behavioral data through the use of augmented reality in existing buildings can increase the user’s sense of realism and therefore obtain more reliable data.

## 4. Conclusions

The most important quantitative results of the representative case relate to the time: the recognition time or interval between the alarm sounding and the user realizing there is an emergency. It took 25% of the users’ time to realize that the sound they heard was a fire alarm.

The average response time or interval between the “recognition time” and the time when the user begins to move to a safe zone is 57 s.

It can be concluded that more than one minute elapses, on average, from the time the alarm sounds until the user decides to start the move to a safe area.

Another interesting fact is that only 6.4% of the participants saw at least one evacuation signal during the move to a safe zone.

In all cases, there is an increase in the users’ heart rate from the moment the fire alarm sounds; there are more head movements, and there is also an increase in the level of anxiety before and after the experience.

This research has shown that it is possible to capture and record the most important variables of human behavior and, therefore, it is possible to analyze decision-making from the moment the fire alarm sounds.

The most important limitation of this study is that it was conducted in a single building.

To date, tests have been carried out on different user profiles, but more tests will be necessary to create models for people movement simulators.

An improvement proposal is the use of augmented reality to particularize cases in specific buildings. Capturing behavioral data by using augmented reality in existing buildings can increase the user’s sense of realism; therefore, we can obtain more reliable data.

Data on human behavior are essential for technicians who design building evacuation protocols, simulation software development companies, and architectural and engineering professionals.

Behavioral data can help to design evacuation procedures for particularly vulnerable groups, such as people with disabilities.

This protocol makes it possible to record behavioral data without the user feeling any sense of threat and to observe the changes that occur once the alarm sounds and the user beings to transition to the safety zone.

To date, tests have been carried out on different user profiles, but more tests will be necessary to create models for people movement simulators.

The results are not valid if the user feels unwell. Another limitation of this method is the high cost associated with the complete development of a realistic scenario.

The use of augmented reality will avoid the use of the platform for travel within the space and will make the experience more realistic.

## Figures and Tables

**Table 1:** Data-collection techniques.

Data-Collection Technique	Real Time	Previous Knowledge of the Fire	Data Collected during the Fire
Surveys	No	No	No
Observations	Yes	Yes	No
Simulation	No	Yes	No
Virtual Reality	Yes	No	Yes

**Table 2:** Hardware specifications of devices used in the current study.

HTC VIVE Series Virtual Reality System	Computer Equivalent or Better	3dRudder Foot Controller	Polar Heart Rate Monitor
Steam VR tracking	Intel Core i7	Free movement	Optical
G-sensor	16 Gb RAM	Spin movement	Bluetooth
Gyroscope	Graphics GTX 1070	Hands-free	ANT+™
Proximity Tracked area. Up to 15 m^2^ Integrated microphone Multifunction trackpad	Windows^®^ 10	Progressive	

**Table 3:** Behavioral variables identified in the protocol.

Behavioral Variables	Parameters
1. Times recorded for the user during the experiment	Recognition time or interval between the time when the alarm sounds and when the user recognizes the emergency. Interval between the recognition time and the time when the user begins to move to a safe zone. Travel time or interval between response time and completion of access to a safe zone. Time during which the user is affected by the existence of smoke.
2. Complete path traveled from the beginning of the alarm to the end	Trajectory during recognition time and response time. Path taken to travel to the safe zone. Trajectory covered while the user is crouching, crawling or squatting
3. User speed (walking, running or crawling) during the entire trajectory	Within the virtual environment, speeds are programmed according to the user profile (age, motor disability, anthropometry, etc.).
4. Emergency signs displayed during travel time	The instant in which the user focuses his or her gaze on an emergency sign is captured.
5. Signal attention	Sudden head movements from the beginning of the experience to the end.
6. Physiological parameters	Heart rate from the beginning of the experience to the end Blood pressure from the beginning of the experience to the end
7. Actions	Opening and closing of doors during evacuation.

**Table 4:** Age of users.

User Age Range	%
<12	26%
12–18	21%
19–35	25%
36–60	27%
>60	1%

**Table 5:** User profile and trait anxiety level before the experience.

Profile	User 1	User 2
Age	35	38
Sex	male	female
Education level	higher	higher
Disability	No	No
Average heart rate before the alarm signal	78	82
Trait anxiety level	4	5

**Table 6:** Behavioral parameters and state anxiety level after the experience.

Behavioral Parameters	User 1	User 2
Recognition time	0.1	0.3
Response time	38	35
Travel time	91	96
Visualization of evacuation signals	0	2
Path	Path1	Path2
Average heart rate 30 s after alarm sounds	83	88
Average heart rate while user crosses the area filled with smoke	104	103
Sudden head movements	31	33
State anxiety level	6	7
Previous experiences in fires	no	no
Experiences like this can help people’s safety	4	4
